# Cognitive impairment six months after ischaemic stroke: a profile from the ASPIRE-S study

**DOI:** 10.1186/s12883-015-0288-2

**Published:** 2015-03-12

**Authors:** Lisa Mellon, Linda Brewer, Patricia Hall, Frances Horgan, David Williams, Anne Hickey, on behalf of the ASPIRE-S study group

**Affiliations:** Department of Psychology, Division of Population Health Sciences, Royal College of Surgeons in Ireland, Dublin, 2 Ireland; Department of Geriatric and Stroke Medicine, Royal College of Surgeons in Ireland, Dublin, 2 Ireland; School of Physiotherapy, Royal College of Surgeons in Ireland, Dublin, 2 Ireland

**Keywords:** Cognition, Cognitive impairment, Stroke rehabilitation, Secondary prevention

## Abstract

**Background:**

Cognitive impairment commonly occurs in the acute phase post-stroke, but may persist with over half of all stroke survivors experiencing some form of long-term cognitive deficit. Recent evidence suggests that optimising secondary prevention adherence is a critical factor in preventing recurrent stroke and the incidence of stroke-related cognitive impairment and dementia. The aim of this study was to profile cognitive impairment of stroke survivors at six months, and to identify factors associated with cognitive impairment post-stroke, focusing on indicators of adequate secondary prevention and psychological function.

**Methods:**

Participants were assessed at six months following an ischaemic stroke as part of the Action on Secondary Prevention Interventions and Rehabilitation in Stroke study (ASPIRE-S), which examined the secondary preventive and rehabilitative profile of patients in the community post-stroke. Cognitive impairment was measured using the Montreal Cognitive Assessment (MoCA).

**Results:**

Two-hundred and fifty-six stroke patients were assessed at six months. Over half of the sample (56.6%) were found to have cognitive impairment, with significant associations between cognitive impairment and female sex (odds ratio (OR) = 1.6, 95% CI 1.01-2.57) and history of cerebrovascular disease (OR = 2.22, 95% CI 1.38-3.59). Treatment with antihypertensive medications (OR = .65, 95% CI .44-.96) and prescription of anticoagulant therapy (OR = .41, 95% CI .26-.68) were associated with reduced likelihood of cognitive impairment, however increasing number of total prescribed medications was moderately associated with poorer cognitive impairment (OR = 1.12, 95% CI 1.04-1.19).

**Conclusions:**

Findings reveal levels of cognitive impairment at 6 months post-stroke that are concerning. Encouragingly, aspects of secondary prevention were identified that may be protective in reducing the incidence of cognitive impairment post-stroke. Neuropsychological rehabilitation post-stroke is also required as part of stroke rehabilitation models to meet the burden of post-stroke cognitive impairment.

## Background

Stroke is the second most common cause of death in the world after ischaemic heart disease [1] and the leading cause of acquired disability, with over half of patients remaining physically dependent following a stroke [2] and approximately two-thirds having some form of neurological impairment at five years post-stroke [3]. Restoration of physical function in stroke is widely researched, with evidence demonstrating significant improvements following physical rehabilitation [4]. However strategies for restoration of cognitive dysfunction receive significantly less attention with cognitive rehabilitation post-stroke arguably the “lost dimension” of stroke rehabilitation. Estimation of the prevalence of post-stroke cognitive impairments is difficult given the range of potential deficits, including memory, attention, and visuospatial ability, thus reported rates have varied from 30-50% [5]. A degree of cognitive impairment is often evident in the immediate aftermath of stroke with many deficits resolving over the initial recovery period [6]. However longitudinal evidence in the United Kingdom [7] has demonstrated the long-term prevalence of cognitive impairment in stroke, with prevalence rates of 22% at five years and 21% at 14 years reported, highlighting the persistent pervasiveness of cognitive deficits. Cognitive impairment can have a significant impact on quality of life and activities of daily living by reducing the degree of independence of the individual [8] and is associated with long-term morbidity and disability [7].

Up to 34% of patients with dementia show significant vascular pathology [9]. Because vascular risk factors are treatable and, in some instances preventable, it may be possible to prevent, postpone, or mitigate vascular cognitive impairment (VCI), as well as the vascular exacerbation of Alzheimer’s disease. In a systematic review and meta-analysis, Pendlebury and Rothwell [5] showed that 10% of patients had dementia before first stroke, 10% developed dementia in the year after first stroke and over one-third of patients developed dementia after recurrent stroke. Emerging evidence suggests that appropriate secondary prevention of recurrent events may reduce the burden of post-stroke cognitive impairment and progression to dementia. The Perindopril Protection against Recurrent Stroke Study (PROGRESS) randomised clinical trial reported that active blood pressure management was associated with a 19% risk reduction in cognitive decline [10]. A recent UK study [11] also suggested that appropriate management of vascular risk factors post-stroke was associated with longer term protective benefits for onset of cognitive impairment. In particular, management of vascular risk factors, including adequate blood pressure control, lipid control and anti-thrombotic therapy were associated with reduced risk of post-stroke cognitive impairment over a 15 year follow-up period.

The cognitive profile of Irish stroke survivors at six months was examined as part of the Action on Secondary Prevention Interventions and Rehabilitation in Stroke study (ASPIRE-S). The ASPIRE-S study was a prospective descriptive study which aimed to address three components of stroke care following discharge from acute hospital care, namely secondary preventive strategies, delivery of community rehabilitative care and ongoing rehabilitative need. The aim of the analysis presented in this study was to comprehensively profile post-stroke cognitive impairment and, in doing so, to determine which factors were associated with cognitive impairment in this stroke survivor cohort.

## Methods

### Study population and procedure

The ASPIRE-S participants were consecutively recruited first ever and recurrent stroke cases in patients of all ages admitted to three large teaching hospitals between October 1st 2011 and September 30th 2012. The North Dublin Population Stroke Study group (NDPSS) [12-14] has previously characterised the stroke population in North Dublin, providing detailed clinical information on incidence and clinical profile of stroke patients in this geographical area of Ireland. The study received ethical approval from the Research Ethics Committees of the three participating hospitals. Participants consented to participate in ASPIRE-S whilst in hospital with an acute stroke, with assent from next-of-kin obtained for patients identified by the medical team as lacking capacity to give informed consent. In-hospital recruitment was conducted by the research team which included a cardiovascular research nurse, a clinical research fellow and research assistant across the three hospital sites. Consented participants were contacted at six months post-stroke to participate in a detailed assessment of secondary preventive and rehabilitative indicators. The assessment by a member of the research team took place either in the participant’s home or in dedicated clinical research facilities at one of the participating hospitals, depending on the participant’s preference.

The study focused on hospital-based case ascertainment, as NDPSS found that over 90% of stroke cases were treated in an acute hospital [12]. Case ascertainment was identified by daily review of referrals to the neurology, geriatric and stroke consultation services for each of the participating hospital sites. The World Health Organisation (WHO) definition of stroke was used to define presenting stroke as “rapidly developing clinical signs of focal or global disturbance of cerebral function, lasting more than 24 hours or leading to death with no apparent cause other than that of vascular origin” [15]. Participants were excluded if the following criteria were identified: diagnosis of subarachnoid haemorrhage, intracerebral haemorrhage or sub-dural and extra-dural haematoma; transient ischaemic attack (TIA); residence outside the geographic area defining the source population; age < 18 years.

### Measures

#### Baseline assessment

Demographic and baseline clinical characteristics were recorded for each participant during in-hospital recruitment including age, sex, cardiovascular risk factors, stroke severity (Scandinavian Stroke Scale (SSS) [16] and stroke sub-type using the Bamford classification [17] and TOAST criteria for stroke aetiology [18]. Pre-stroke functional dependency was assessed retrospectively and at 72 hours, using the Modified Rankin Scale (mRS) [19], a commonly used measure of global disability used as a functional outcome measure in stroke populations [20]. All members of the team received standardised training in the administration of all assessment measures to minimise measurement variability.

### Six-month assessment

#### Ongoing rehabilitative need and functional dependency

A number of assessments were performed to assess level of disability and rehabilitative need.

1. *Cognitive function* was assessed using the Montreal Cognitive Assessment (MoCA) [21]. The MoCA was developed for use in the detection of Mild Cognitive Impairment (MCI), and screens for the common domains of impairment in MCI. Short-term memory recall, delayed recall, visuospatial abilities, executive functions, phonemic and syntactic fluency, verbal abstraction, serial subtraction, attention, concentration and working memory and orientation to time and place are all examined in a 30-point test which can be administered in approximately 10 minutes [21]. The original MoCA publication sets the cut-off threshold for cognitive impairment as a MoCA score of less than 26. The MoCA has been reported as a valid measure for screening for cognitive impairment and dementia in stroke cohorts [22], demonstrating higher sensitivity than the Mini Mental State Examination (MMSE), at the cost of lower specificity [22,23].
2. *Functional status* was assessed using the Modified Rankin Scale (mRS) [20] with higher scores indicating greater functional impairment.
3. *Psychological function* was assessed using a number of measures included in a self-completion questionnaire which was posted to participants in the week preceding the ASPIRE-S assessment. The Hospital Anxiety and Depression Scale (HADS) was included in the self-completion questionnaire, a widely used scale for assessment of mood disorder, containing two dimensions for anxiety and depression [24]. The range of scores for each dimension is 0–21, with a score of greater than 7 indicative of probable or possible presence of symptoms of mood disorder [25]. The HADS has been validated for use in stroke cohorts [26]. The Vulnerable Elders Scale (VES) was also completed by the participant as part of the self-completion questionnaire. The VES is a 13-item measure, with scores greater than a cut-off of 3 indicating vulnerability and increased risk for mortality in the subsequent two years [27]. Post-stroke health-related quality of life (HRQoL) was assessed using the Stroke Specific Quality of Life Scale (SSQoL) [28], a 35-item scale which examines seven domains of quality of life, namely physical function, mood, role function, vision, language, thinking and energy. The maximum score is 175, with higher scores indicating better self-reported HRQoL.

The HADS, VES and SSQoL were included in a postal questionnaire which stroke survivors received one week prior to the ASPIRE-S home assessment. During the ASPIRE-S home assessment, participants were provided with the opportunity to clarify any questions presented in the questionnaire, and ASPIRE-S researchers ensured completeness of the questionnaire.

#### Secondary prevention

A number of clinical efficacy markers were recorded during the six-month assessment, including resting blood pressure using a digital OMRON M6 (Intellisense™) Dual Check System, International Normalised Ratio (INR) control (average of last three INR readings) medications, medication adherence, and carbon monoxide assessment for participants documented as smokers at baseline. Pharmacological secondary prevention was examined through fasting venous blood analysis of serum total cholesterol, high-density lipoprotein cholesterol, low-density lipoprotein cholesterol, triglycerides, fasting glucose and HbA1c (diabetics only). All participants were fitted with a 24-hour ambulatory blood pressure monitor where possible.

### Statistical analysis

Descriptive statistics and non-parametric comparisons using χ^2^ test and Fisher’s Exact test for categorical variables and Kruskal Wallis test for continuous variables were used to examine the association between cognitive impairment (MoCA <26) and demographic and clinical characteristics. The degree of association between individual variables and cognitive impairment was estimated using the odds ratio (OR) and 95% confidence interval (CI). Multivariable analysis was adjusted for age, sex and stroke severity as these variables are highly correlated with stroke outcome [29]. Considerations for modelling sample size were applied to avoid over-fitting of the models and subsequent production of biased estimates of effect size [30,31]. Statistical analysis using the cluster option in Stata 12.0 provided Huber-White-robust standard error estimates as data were collected across three differing hospital sites. Targets for secondary preventive indicators (blood pressure, glucose, cholesterol) were calculated using the European guidelines on cardiovascular disease prevention in clinical practice 2012 [32] and recommendations from the EUROASPIRE III stroke study [33]. Raised blood pressure was defined as systolic blood pressure ≥140 mmHg and or diastolic blood pressure ≥90 mmHg. Raised cholesterol levels were defined as total cholesterol concentration ≥4.5 mmol/L, and/or LDL cholesterol ≥2.5 mmol/L, and impaired fasting glycaemia was defined as fasting glucose <7 mmol/L [33].

## Results

Three hundred and two stroke cases were recruited to the ASPIRE-S study at baseline. Approximately 52% of all referred cases were ineligible for study inclusion at baseline, with the most common reasons for non-inclusion recorded as patient diagnosed as TIA following MRI imaging (17.9%), or too medically unwell during hospital admission (16%). Of the 302 stroke cases recruited at baseline, 256 assessments were completed at six months post-stroke (84.8%). Reasons for non-completion of follow-up were death in preceding six months (n = 9), declined participation (n = 22), development of serious illness (n = 6), final discharge diagnosis as not stroke (n = 4) and uncontactable (n = 5). The mean age was 69 years (±12.9; median 71 years, IQR = 61-78) at time of stroke onset, with 96.4% of patients functionally independent prior to the stroke, defined by mRS <3. Median stroke severity score on the SSS was 52 (IQR 45–58). Prevalence of functional dependence at six months post stroke (mRS ≥3) was 30.8% (N = 79), reduced from 47.5% (n = 121) at 72 hours post-stroke.

### Cognitive performance at six months

MoCA assessment was completed for 226/256 participants (88.2%). The mean MoCA score was 23.5 (SD ± 4.9), with scores ranging from 7 to 30. Performance on each of the MoCA sub-domains and associated confidence intervals are presented in Table 1. Poorest performance was evident in recall (Mean = 2.53; SD ± 1.6; range 0–5), and visuospatial and executive functioning (Mean = 3.53; SD ± 1.5; range 0–5). When the standard MoCA cut-off of <26 for cognitive impairment was applied to the sample, 128 participants (56.6%) scored in the impaired range on MoCA at six months post-stroke. A higher proportion of females were categorised as cognitively impaired. The presence of a number of risk factors had an association with cognitive impairment in univariate analysis, namely history of a previous cerebrovascular event, atrial fibrillation and carotid stenosis (See Table 2). The burden of cognitive impairment was further explored by lowering the threshold for cognitive impairment on MoCA scoring to <23 [23]. The prevalence of cognitive impairment in the sample using this cut-off was 92/226 (41%), indicating a pervasive level of impairment in the sample.

**Table 1:** **Mean domain scores for MoCA**

**MoCA domain**	**Scoring range**	**Mean score**	**95% CI**
Visiospatial and executive function	0-5	3.53	3.33-3.73
Naming	0-3	2.62	2.52-2.72
Attention	0-6	5.23	5.06-5.40
Language	0-3	2.22	2.10-2.34
Abstraction	0-2	1.72	1.64-1.79
Delayed recall	0-5	2.53	2.32-2.74
Orientation	0-6	5.65	5.54-5.76
Total MoCA score	0-30	23.53	22.88-24.18

CI, Confidence Interval.

**Table 2:** **Demographic and clinical details at baseline and at six months for cognitively impaired and non-cognitively impaired stroke survivors**

	**Cognitive impairment**	**No cognitive impairment**			
	**(n = 128; MoCA < = 25)**	**(n = 98; MocA >26)**			
	**N (%)**	**N (%)**	***P*** **value**	**Univariate OR**	**95% CI**
*Age (Mean,* ±*SD)*	71.8 (11.4)	63.2 (13.1)	0.001	1.06	1.02-1.09
*Sex*					
Female	63 (67.7)	30 (32.3)	<0.001	2.19	1.97- 2.45
*Health Insurance status as proxy SES*					
Public patient	102 (65.8)	53 (34.2)			
Private patient	26 (36.6)	45 (63.4)	0.001	.33	.14-.63
*Baseline risk factors*					
Previous TIA/Stroke	41 (74.5)	14 (25.5)	<0.001	2.83	1.6-4.9
Heart disease	42 (63.6)	24 (36.4)	0.91	1.5	.63-3.6
Hypertension	76 (60.3)	50 (39.7)	0.055	1.4	.99-1.98
Diabetes	24 (64.9)	13 (35.1)	0.059	1.5	.98-2.31
Hypercholesterolemia	65 (58.6)	46 (41.4)	0.23	1.16	.91-1.49
Smoker	34 (52.3)	31 (47.7)	0.18	.78	.54-1.12
Atrial fibrillation	53 (63.9)	30 (36.1)	<0.001	1.6	1.26-1.04
Carotid stenosis	21 (61.8)	13 (38.9)	<0.001	1.28	1.14-1.45
*Bamford classification*					
TACS	6 (66.7)	3 (33.3)	0.67	1.56	.21-11.63
PACS	51 (55.4)	41 (44.6)	0.7	.92	.1-1.4
POCS	36 (53.7)	31 (46.3)	<0.001	.84	.77-.92
LACS	33 (62.2)	20 (37.8)	0.002	1.35	1.12-1.64
*TOAST classification*					
Large artery atherosclerosis	19 (52.8)	17 (47.2)	0.54	.83	.46-1.49
Cardioembolism	52 (59.8)	35 (40.2)	0.22	1.23	.88-1.72
Small vessel occlusion	12 (42.9)	16 (57.1)	<0.001	.53	.45-.63
Other determined aetiology	4 (30.8)	9 (68.2)	<0.001	.31	.23-.45
Undetermined aetiology	41 (66.1)	21 (33.9)	0.3	1.72	.61-4.89
Stroke severity (SSS scale; *Median, IQR*)	51 (42–57.5)	56 (49–58)	<0.001	.92	.89-.94
*Functional dependency*					
Pre-stroke function (mRS; *Median, IQR)*	0 (0–0)	0 (0–0)	0.02	2.07	1.08-3.95
Function at 72 hours (mRS; *Median, IQR)*	3 (1–4)	1 (0–3)	<0.001	1.56	1.41-1.71
Function at six months	2 (1–3)	1 (1–2)	<0.001	1.76	1.32-2.34
(mRS; *Median, IQR)*					
VES (> = 3)	71 (73.2)	26 (26.8)	0.01	5.8	1.51-22.53
(n = 200)					
HADS-D (>7)	24 (66.7)	12 (33.7)	0.16	2	.76-5.26
(n = 190)					
HADS-A (>7)	27 (50.9)	26 (49.1)	.98	0.95	.64-1.52
(n = 190)					
SSQoL (*Median, IQR)*	135 (110–153)	152 (135–170)	<0.001	0.97	.96-.98
(n = 173)					

OR, odds ratio; CI, confidence interval; SD, standard deviation; SES, socioeconomic status; TIA, transient ischaemic attack; TACS, total anterior circulation stroke; PACS, partial anterior circulation stroke; POCS, posterior circulation stroke; LACS, lacunar stroke; SSS, stroke severity; mRS, modified Rankin Scale; VES, Vulnerable Elders Survey; HADS-D, Hospital Anxiety and Depression Scale- Depression; HADS-A, Hospital Anxiety and Depression Scale- Anxiety; SSQoL, Stroke Specific Quality of Life.

The association between sex and presence of cognitive impairment was modestly attenuated in multivariable analysis adjusted for age and stroke severity, with women more likely to have cognitive impairment than men (OR = 1.6, 95% CI 1.01-2.57). History of a previous cerebrovascular event was also related to an increased likelihood of cognitive impairment (OR = 2.2, 95% CI1.38-3.59). Posterior circulation (OR = 1.86, 95% CI 1.84-1.89) and lacunar strokes (OR = 1.72, 95% CI 1.38-2.14) were also more likely to score in the impaired MoCA range when adjusting for age and stroke severity (See Table 3).

**Table 3:** **Demographic and clinical factors associated with cognitive impairment at 6 months post-stroke**

	Adjusted OR*	***P-*** **value**	**95% CI**
Sex (female)	1.6	0.05	1.01-2.57
Age	1.06	0.003	1.02-1.11
Stroke severity (SSS scale)	.92	0.003	.88-.97
Insurance	.23	0.002	.09-.59
History of TIA/Stroke	2.22	0.001	1.38-3.59
History of atrial fibrillation	1.06	0.83	.62-1.82
History of carotid stenosis	.99	0.95	.76-1.29
POCS	1.86	<0.001	1.84-1.89
LACS	1.72	<0.001	1.38-2.14
Small artery occlusion	.46	<0.001	.43-.5
Stroke of determined aetiology	.99	1.00	.65-1.54
Stroke of undetermined aetiology	1.64	0.4	.51-5.2
Functional dependency at 6 months (mRS ≥ 3)	1.06	0.83	.63-1.79

*adjusted for age, sex and stroke severity.

OR, odds ratio; CI, confidence interval; SSS, stroke severity; TIA, transient ischaemic attack; POCS, posterior circulation stroke; LACS, lacunar stroke; mRS, modified Rankin Scale.

Key secondary preventive targets were assessed for their association with cognitive impairment (Table 4). Increasing number of prescribed medications was associated with higher likelihood of presence of cognitive impairment (OR = 1.12, 95% CI 1.04-1.19). Polytherapy with >2 antihypertensive medications (OR 0.67, 95% CI 0.46-0.96) and an anticoagulant (OR = .41, 95% CI .26-.68) were shown to have a protective effect for reduced likelihood of cognitive impairment. Antiplatelet therapy was also associated with protection against cognitive impairment (OR = .38, 95% CI .13-1.11), however this effect did not reach statistical significance (Table 4). A moderate association was evident between uncontrolled total cholesterol level and increased likelihood of cognitive impairment (OR = 1.86, 95% CI .91-3.82), with the observed *p-*value approaching statistical signifiance.

**Table 4:** **Secondary preventive factors associated with cognitive impairment at 6 months post-stroke**

	Adjusted OR*	***P-*** **value**	**95% CI**
Blood pressure not at target (>140/90)	1.02	0.93	.72-1.4
Fasting glucose not at target <7 mmol/L)	1.11	0.73	.62-1.96
Serum total cholesterol not at target (>4.5 mmol/L)	1.86	0.091	.91-3.82
Number of medications	1.12	0.001	1.04-1.19
Antihypertensive	1.22	0.44	.74-2.01
Polytherapy antihypertensive (<2 vs. > = 2)	.67	0.03	.46-.96
Antiplatelet	.38	0.078	.13-.68
Anticoagulant	.41	<0.001	.26-.68
Statin	.75	.59	.28-2.04

*adjusted for age, sex and stroke severity.

OR, odds ratio; CI, confidence interval.

Measures of psychosocial well-being were assessed for their relationship to cognitive impairment. Those identified as vulnerable on the VES, characterised by lower VES scores, were more likely to have cognitive impairment at 6 months (*β* = −.39, *p =* 0.01, 95% CI -.58 - -.22*).* Better quality of life on the self-reported SSQoL scale also was associated with higher MoCA scores (*β* = .05, *p <* 0.001, 95% CI .03-.08*)*. No relationship was found between scores in the depressed or anxious range on HADS and cognitive impairment (Table 5).

**Table 5:** **Psychological well-being factors associated with cognitive impairment at 6 months post-stroke**

	**Coefficient (β)**	***P-*** value*	**95% CI**
Vulnerability (VES)	-.39	0.01	-.58 - -.22
SSQoL	.05	0.009	.03-.08
HADS-Depression	.32	0.09	-.12-.76
HADS- Anxiety	-.04	0.81	-.70-.62

*adjusted for age, sex and stroke severity.

OR, odds ratio; CI, confidence interval; VES, Vulnerable Elders Survey; SSQoL, Stroke Specific Quality of Life Scale; HADS, Hospital Anxiety and Depression Scale.

## Discussion

This study profiled the prevalence of cognitive impairment in a sample of Irish stroke survivors, and investigated the relationship between cognitive impairment and demographic, secondary preventive, clinical, and psychosocial factors. Findings from this study indicate that over 50% of patients exhibited cognitive impairment six months post-stroke. This finding is similar to previous reported rates of cognitive impairment, which can vary from approximately 30%-74% depending on follow-up time and stroke subtype [34,35]. The six month measurement window presented in this study can be considered as relatively recent following stroke occurrence, however longitudinal findings from the South London Stroke Register indicate a significant ongoing prevalence of cognitive impairment, with a rate of 21% evident up to 15 years post-stroke [7]. In this analysis, females, those with a history of cerebrovascular disease and those experiencing a posterior circulation or lacunar infarct were most likely to exhibit cognitive impairment. We also found that presence of cognitive impairment was associated with increased likelihood of patient vulnerability and poorer quality of life. Given this high prevalence of cognitive impairments post-stroke and its contribution to dementia progression and poor psychological outcome, cognitive rehabilitation is a required component of stroke rehabilitation. The current evidence for the effectiveness of cognitive rehabilitation in stroke is sparse, with very few studies using randomised designs [36].

Findings from this study support emerging evidence for the important role of secondary preventive strategies in prevention of post-stroke cognitive decline and transitions to dementia [5,11]. Results showed that polytherapy for hypertension, i.e. taking two or more antihypertensive medications, demonstrated a protective effect for the presence of cognitive impairment at six months post stroke. Prescription of both anticoagulant and antihypertensive therapies also demonstrated a protective effect, suggesting that appropriate pharmacotherapy post-stroke may contribute towards slowing of cognitive decline post-stroke in addition to prevention of further stroke. Previous studies have reported the protective association between optimal risk factor control and reduced cognitive impairment, with protective effects reported up to ten years post-stroke in a UK population-based study [11]. There was an observed trend in this analysis between uncontrolled total cholesterol and the presence of cognitive impairment, although did not reach statistical significance in this instance. This relationship has been confirmed in previous studies. A Finnish longitudinal cardiovascular risk factors study found that mid-life serum cholesterol levels were associated with an increased risk of vascular dementia and Alzheimer’s disease in later life, even after adjustment for statin therapy [37]. Serum cholesterol control is a key component of basic secondary preventive strategies in cardiovascular conditions, but still remains a challenge.

Interestingly, there appeared to be a relationship between prescribed complex medication regimens and presence of cognitive impairment in this sample, with greater numbers of prescribed medications related to higher likelihood of cognitive impairment. Cognitive impairment has been cited as a common barrier to successful medication management in the elderly [38], and polypharmacy in older populations is a known risk factor for morbidity and mortality [39]. Important interactions between polypharmacy, cognitive impairment and adverse drug reactions have been reported in the literature [40]. Correct adherence requires both time, effort and understanding on behalf of the patient, thus as the complexity of the regimen is increased, so also is the burden on the patient [41]. Managing a complex medication regimen whilst exhibiting signs of post-stroke cognitive impairment is likely to increase the risk of non-adherence, resulting in deleterious effects on secondary prevention targets, further cognitive decline, substantially increased risk of recurrent stroke, with its associated implications for subsequent dementia. Non-adherence to medications has been found to be common for patients with cardiovascular diseases, with a progressive decline in adherence to prescribed secondary preventive medications evident with the passage of time [42], and is associated with increased risk of morbidity and mortality. In the context of stroke, cognitive impairment is likely to interfere with patients’ capacity to adhere to their medication regimen.

The NICE guidelines recommend that each stroke patient should receive cognitive screening, regardless of stroke severity, within 6 weeks of their stroke, in order to identify cognitive deficits, including higher level impairments that may impede normal activities of daily living [43]. Evaluation of current practices for assessment of cognitive impairment in the in the UK revealed that up to 85% of healthcare professionals routinely screen for cognitive impairment, however significant heterogeneity exists in method of assessment [44]. Little Irish data exists to demonstrate that post-stroke cognition is routinely tested, and the Irish National Cardiovascular Health Policy has highlighted that the lack of inclusion of cognitive rehabilitation as part of standard stroke rehabilitation is of prominent concern [41]. A lack of standardisation in definition, evaluation and the timing of assessment for post-stroke cognitive impairment exists [45]. The MoCA in particular is subject to ongoing debate. It is argued that the universal cut-off score of less than 26 may over diagnose the existence of true cognitive impairment [46], and cut-offs of less than 23 have been suggested as having better clinical utility in stroke [46,47]. Its brevity is useful in the context of stroke where patient fatigue may be prevalent, however, it is a brief screening tool for mild cognitive impairment (MCI) and therefore does not capture all post-stroke deficits. In-depth neuropsychological testing, such as the NINDS [48], which accounts for a wide range of potential deficits and abilities is a recommended form of assessment [48].

### Limitations

The study was an observational cohort study, with no matched non-stroke group available for comparison of cognitive outcomes. Cognitive impairment was measured at only one time point during the stroke recovery period, and pre-stroke cognitive function was not measured. Although the observed prevalence of impairment at six months was high, pre-existing cognitive decline may have been evident in some cases, given the age profile of the sample and the finding that a quarter of the sample had experienced a previous cerebrovascular event. It is suggested that a measure such as the Informant Questionnaire for Cognitive Decline in the Elderly (IQCODE) [49] be incorporated into research designs were possible to capture an indicator of pre-stroke cognitive function. Patients with TIA were excluded from the present analysis. It has been demonstrated that approximately a third of patients with TIA can exhibit impairment in at least one domain of cognitive function following a TIA [50], therefore it may be important to screen for cognitive changes in minor cerebrovascular events in addition to confirmed stroke cases [51]. The aim of this study was to provide as yet unknown data on post-stroke cognitive impairment in an Irish cohort, and focused on global cognitive impairment. Further exploration of the relevance of impairments in different domains for the development of appropriate rehabilitation interventions is needed. Finally, the findings of study are limited to stroke cases presenting to hospital. Population studies in Ireland indicate that 90% of stroke cases are treated in acute care, however those who do not present to hospital may differ in stroke severity, and thus may have very different cognitive outcomes. Future work should attempt to capture outcomes for stroke cases treated exclusively in the community.

## Conclusions

The predicted international population age profile shift will result in more individuals at risk of stroke, an increased burden on health and community services for stroke, and a greater cost to national economies. This paper reports findings from the ASPIRE-S study relating to prevalence of cognitive impairment in a cohort of patients 6 months post-stroke, and found a high prevalence of cognitive impairment six months post stroke. Addressing cognitive impairment is of significant importance, both for the individual with stroke who is prevented from resuming engagement in normal life by impairments in cognition, for patients’ families and for wider society who ultimately support the care of those with post-stroke cognitive impairment. Appropriate targeting of secondary prevention control and specific cognitive rehabilitation has the potential to prevent recurrent stroke and the progression from cognitive impairment to dementia in people with stroke.
